# Hybridization between two high Arctic cetaceans confirmed by genomic analysis

**DOI:** 10.1038/s41598-019-44038-0

**Published:** 2019-06-20

**Authors:** Mikkel Skovrind, Jose Alfredo Samaniego Castruita, James Haile, Eve C. Treadaway, Shyam Gopalakrishnan, Michael V. Westbury, Mads Peter Heide-Jørgensen, Paul Szpak, Eline D. Lorenzen

**Affiliations:** 1 Natural History Museum of Denmark, University of Copenhagen, Øster Voldgade 5-7, 1350 København K, Denmark; 2 Greenland Institute of Natural Resources, Strandgade 91,2, DK-1401 Copenhagen K, Denmark; 3 Department of Anthropology, Trent University, 1600 West Bank Drive, K9L0G2 Peterborough, Ontario Canada

**Keywords:** Stable isotope analysis, Speciation, Evolutionary biology

## Abstract

In 1990, a skull from a morphologically unusual Monodontid was found in West Greenland and collected for the Natural History Museum of Denmark, University of Copenhagen. From its intermediate morphology, the skull was hypothesized to be a beluga/narwhal hybrid. If confirmed, the specimen would, to our knowledge, represent the sole evidence of hybridization between the only two toothed whale species endemic to the Arctic. Here we present genome-wide DNA sequence data from the specimen and investigate its origin using a genomic reference panel of eight belugas and eight narwhals. Our analyses reveal that the specimen is a male, first-generation hybrid between a female narwhal and a male beluga. We use stable carbon and nitrogen isotope analysis to investigate the dietary niche of the hybrid and find a higher *δ*^13^C value than in both belugas and narwhals, suggesting a foraging strategy unlike either parental species. These results further our understanding of the interaction between belugas and narwhals, and underscore the importance of natural history collections in monitoring changes in biodiversity. In addition, our study exemplifies how recent major advances in population genomic analyses using genotype likelihoods can provide key biological and ecological insights from low-coverage data (down to 0.05x).

## Introduction

**Figure 1:**
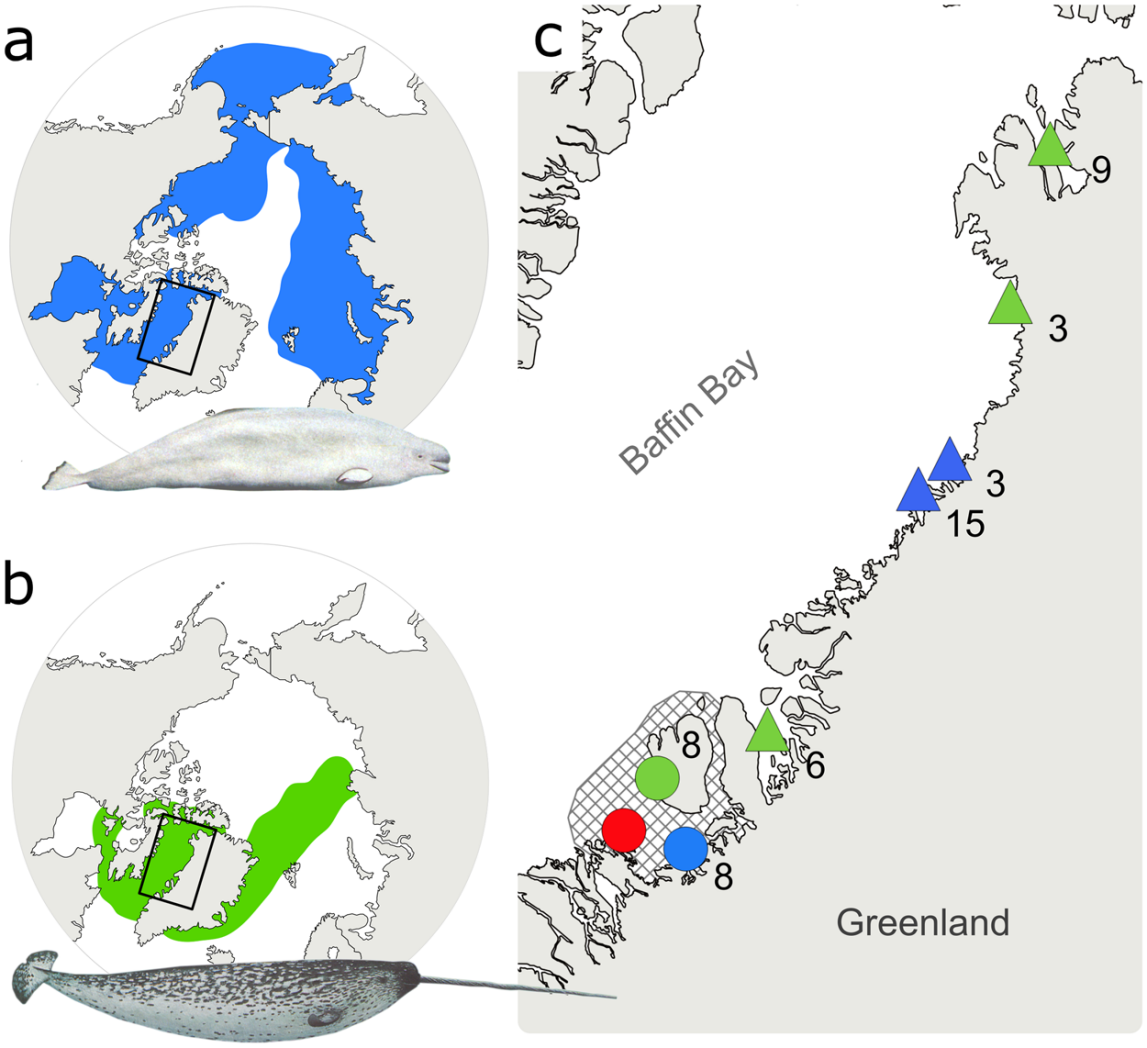
Distribution ranges of (**a**) belugas and (**b**) narwhals, and (**c**) insert map of sampling localities of the beluga (blue) and narwhal (green) used in the study. Circles represent tissue samples used in the genomic analyses, triangles represent the skulls used in the stable isotope analysis. The collection site of specimen MCE1356 is indicated with a red circle. Sample sizes at each locality are indicated. Hatched grey area in Fig. 2c represents Disko Bay and adjacent waters. Whale illustrations in (**a**) and (**b**) by Larry Foster.

Of the 89 extant cetacean species, only three are found in Arctic waters year-round. Belugas, or white whales (*Delphinapterus leucas*), and narwhals (*Monodon monoceros*), are medium-sized toothed whales and the sole representatives of the Monodontidae family. The Monodontids constitute a characteristic component of Arctic ecosystems along with the bowhead whale (*Balaena mysticetus*), a baleen whale. Belugas have a disjunct distribution, with populations in both Pacific and Atlantic sectors of the Arctic, with a hiatus in the Greenland Sea (Fig. 1a)^1^. Narwhals have a more limited distribution in the Atlantic sector (Fig. 1b)^2^. Based on a combination of mitochondrial and nuclear genes, the two species are estimated to have diverged ~5 MYA^3^, and a recent study of their nuclear genomes showed that subsequent gene flow between belugas and narwhals ceased between 1.25 and 1.65 MYA^4^.

**Figure 2:**
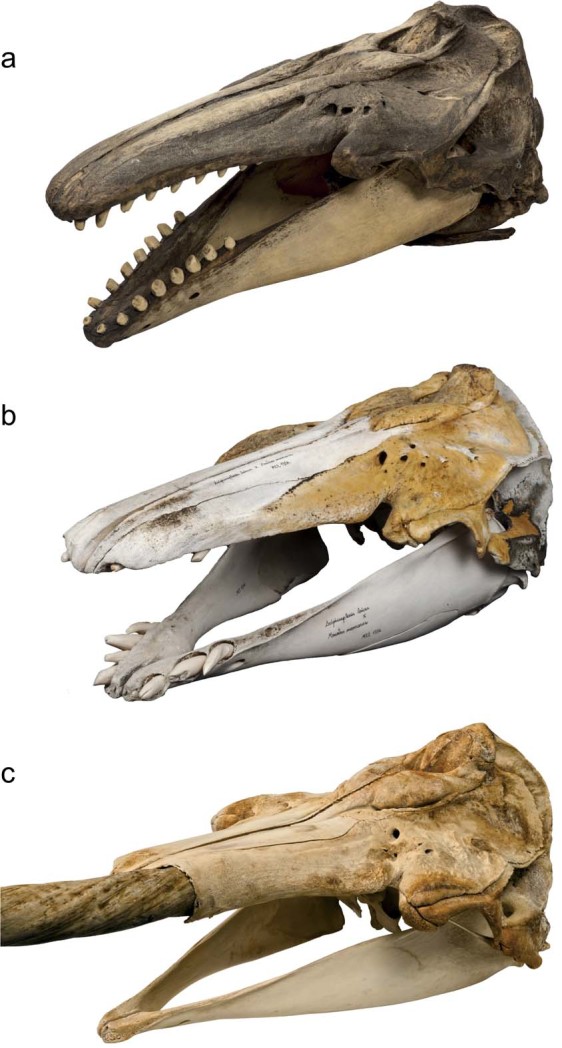
Skull morphology of (**a**) beluga, (**b**) MCE1356, and (**c**) narwhal. Photos: Mikkel Høegh Post.

Belugas and narwhals are similar-sized whales (3.5–5.5 meters), and both exhibit inherited migratory patterns following the annual break up and formation of sea ice^5,6^. For both species, mating takes place in spring during the sea ice break up, as the whales are heading towards their summering grounds. Due to the inaccessibility of the whales in the pack ice during this period, their mating is not well understood. However, both species have similar breeding and nursing behavior, and females may calve every two to four years^7^. Nonetheless, despite their similarities, belugas and narwhals differ in several key aspects. Adult belugas have white skin^1^ whereas narwhals have spotted skin with brown, black, grey and white flecks (Fig. 1)^2^. Belugas have up to 40 teeth, whereas narwhals have no teeth in the lower jaw and males have an elongated front tooth that protrudes through their upper lip and can grow up to 2.5 meters in length (Fig. 2)^8^. The two species differ in prey selection and diving capabilities; belugas mainly feed on fish down to 500 meters^9^, whereas narwhals have the capacity to feed on fish and squids at depths >800 meters^10^.

In 1990, an unusual whale skull was collected from a hunter on an island in Disko Bay, West Greenland (Fig. 2). Using 12 morphological measurements as well as teeth counts, the skull was quantitatively compared to a reference panel of beluga and narwhal skulls^8^. Although results showed that the skull was larger than those of belugas and narwhals, the measurements revealed that the skull’s relative dimensions were intermediate, in particular the dentition, which showed longitudinal grooves and horizontal orientation of some teeth, resembling the tusk and the vestigial tooth of narwhals^8^ (Supplementary Fig. S1). A hunter caught the whale during a subsistence hunt, and in the near vicinity were two similar-looking whales (both of which were also harvested, but no samples were preserved). All reportedly had an evenly grey coloration and pectoral flippers shaped like those of belugas, and a tail shaped like that of a narwhal. The skull was assumed to belong to a fully grown individual due to the fusion of its premaxilla-maxilla and its large size, and was therefore initially described as either a beluga/narwhal hybrid or an anomalous beluga^8^.

The skull is stored at the Natural History Museum of Denmark, University of Copenhagen (specimen ID MCE1356, Fig. 2b). It is to our knowledge the only evidence of potential hybridization between belugas and narwhals. To investigate whether MCE1356 indeed represents a hybrid, we analyzed genome-wide DNA sequence data retrieved from the specimen against a genomic reference panel of eight belugas and eight narwhals sampled from the same area in West Greenland. In addition, we used stable carbon and nitrogen isotope analysis of bone collagen extracted from the specimen and a reference panel of 18 belugas and 18 narwhals also collected from West Greenland, to investigate the dietary niche of the individual relative to either putative parental species.

## Methods

### Sampling

We analyzed (i) genome-wide DNA data extracted from teeth of the skull (MCE1356, Fig. 2b, Supplementary Fig. S1) and from tissue samples of eight belugas and eight narwhals sampled in Disko Bay, West Greenland, and (ii) stable carbon and nitrogen isotopic compositions of bone collagen from MCE1356 and a reference panel of 18 belugas and 18 narwhal skulls from West Greenland (Fig. 1c). Tissue samples (skin) were stored in 96% ethanol. The samples were collected by scientists from the Greenland Institute of Natural Resources under the general permit for biological sampling of the Inuit from the Greenland Government and exported to Denmark under CITES permit IM 0401-897/04, IM 0721-199/08, IM 0330-819/09 and 116.2006. Sample information is detailed in Supplementary Table S2.

### DNA extraction, library preparation and sequencing

#### Tissue samples

DNA from tissue samples was extracted using the Qiagen Blood and Tissue Kit following the manufacturer’s protocol. The DNA was fragmented using the Covaris M220 Focused-ultrasonicator to create ~350–550 base pair (bp) fragment lengths. Libraries were built from the fragmented DNA extracts using Illumina NeoPrep following the NeoPrep Library Prep System Guide applying default settings. PCR amplification, quantification, and normalization were all carried out by the NeoPrep Library Prep System. The libraries were screened for size distribution on an Agilent 2100 Bioanalyzer and pooled in equimolar ratios before sequencing on an Illumina HiSeq 2500 with 80 bp SE technology.

#### Tooth/bone samples

Unlike the tissue samples, old bone and teeth samples have relatively low DNA concentrations, which necessitates different extraction and library build protocols. Approximately 0.5 g of bone powder from five teeth and one bone shard from specimen MCE1356 was drilled using a hand-held Dremel. DNA was extracted from the tooth/bone powder in a dedicated ancient DNA clean laboratory at the Natural History Museum of Denmark, University of Copenhagen, using the extraction buffer described in^11^ with the addition of a 30 minute predigest stage^12^. Instead of using Zymo-Spin V columns (Zymo Research), the extraction buffer was concentrated using Amicon Ultra 30 kDa Centrifugal Filter Units and further concentrated and cleaned using Qiagen Minelute tubes. The purified extracts were then built into Illumina libraries following the protocol described by^13^. We used qPCR to check that the library build was successful, to select which libraries to sequence, and to calculate the appropriate number of PCR cycles required to sufficiently amplify each library without causing overamplification. In total, four libraries were amplified with unique 6 bp indexes, and screened for endogenous content on the Illumina MiSeq platform using 250 bp SE sequencing. The best libraries were then re-sequenced on the Illumina HiSeq 2500 platform using 80 bp SE technology.

### Bioinformatic analysis

All mapping and DNA damage analyses were performed within the Paleomix pipeline 1.2.12^14^. Reads were trimmed with AdapterRemoval 2.2.0^15^ using default settings except minimum read length which was set to 25 bp. Reads were inspected using FastQC and aligned with BWA^16^ applying the Backtrack algorithm and disabling the starting seed length. If reads mapped to multiple positions or had mapping quality scores (MAPQ score from BWA) less than 30, they were removed using SAMtools^17^. Sequence duplicates were removed using MarkDuplicates from Picard (available from: http://broadinstitute.github.io/picard) and the final alignment was realigned around indels using GATK^18^. Deamination of cytosine to uracil in specimen MCE1356 was assessed using the output from mapDamage v2.0.6^19^. MapDamage results did not show any clear signal of deamination in the sequences (Supplementary Fig. S3).

### Mitochondrial analysis

To determine the maternal lineage of MCE1356, DNA sequencing reads were mapped to the beluga (GenBank accession: KY444734) and narwhal (GenBank accession: NC_005279) mitochondrial reference genomes and mean coverage was compared. Reads from the eight beluga and eight narwhal samples that comprised the reference panel, were mapped to their respective mitochondrial reference genomes. We constructed mitochondrial consensus sequences of MCE1356 and the 16 reference panel samples with regions covered by more than five reads using BEDtools^20^, SAMtools^17^ and GATK^18^. We created two sequence alignments using ClustalW^21^, applying default settings, which included the 16 reference panel samples and either the version of MCE1356 mapped to the beluga mitochondrial reference genome or the the version of MCE1356 mapped to the narwhal mitochondrial reference genome. We used the two alignments to construct median-joining haplotype networks^22^ using Popart 1.7^23^ (available from: http://popart.otago.ac.nz), excluding any sites with indels or missing data. Subsequently, both post-mapping coverage and the two haplotype networks were used to determine the species of MCE1356′s maternal lineage.

### Nuclear DNA analyses

We mapped the DNA sequencing reads from all samples to the killer whale (*Orcinus orca*) reference genome (GCA_000331955.2). A high-quality beluga whale genome was recently published^24^, but mapping to one of the two potential parental species could create biases in the analyses. Hence we mapped the reads to the killer whale genome, as it is well assembled and killer whales are still relatively closely related to belugas and narwhals (divergence time of 12 MYA)^25^, yet distant enough to minimize the risk of introgression that would complicate our analyses.

For all further filtering we used ANGSD v0.923^26^, a software package that uses genotype likelihoods instead of called genotypes, which is particularly useful when analysing low-coverage NGS data. We used the SAMtools^17^ method implemented in ANGSD to estimate genotype likelihoods, and inferred major and minor alleles directly from the genotype likelihoods using a maximum likelihood approach as described in^27^.

To visualise the mapped data, we plotted the read depth distribution from all individuals, excluding sites with more than two alleles and sites with a Phred score below 25. Visualising the data from all individuals combined enabled us to estimate the mean read depth of 4.14x and identify sites with elevated read depth. Such sites were more likely to come from paralogs and repetitive regions of the genome. The dataset was visually inspected and further filtered, excluding all sites with read depth greater than nine (6.9% of sites). In ANGSD, SNPs were called based on their allele frequencies. The minor allele frequency (MAF) was estimated from the genotype likelihoods and a likelihood ratio test was used to determine if the MAF was different from zero. If the *p* value from the likelihood ratio test was <1e-4 the site was considered polymorphic and retained in the dataset. Applying this SNP filter meant that no sites with less than four reads were retained, as sites covered by fewer reads could not obtain *p* values this low. These filters were applied to a data set including all 17 samples, and a dataset without MCE1356.

To determine whether MCE1356 was a beluga/narwhal hybrid, we further filtered the dataset of 17 individuals, excluding sites with no reads in MCE1356. At this point the mean read depth of MCE1356 was only 1.15x, so in order to ensure that we were not analyzing multicopy loci, we excluded sites covered by more than one read in MCE1356.

Our aim was to compare the alleles found in MCE1356 to the alleles in the reference panel, so we estimated the probability of assigning the allele found in MCE1356 to the wrong parental species given different reference panel minimum unique read depths and allele frequencies. Unique read depth was defined as the number of reads covering a specific site, where all reads came from a unique individual. This probability *P* was calculated as in Equation 1:

1 $$P={f}_{(ps)\,}^{\,ur{d}_{(psp)}}\times \,(1-{f}_{(ps)})$$

Where *f*_(*ps*)_ is the allele frequency in the parental species, and *urd*_(*psp*)_ is the species specific unique read depth in the reference panel.

The parental species allele frequency giving the highest probability *f*_(ps-max)_ could be described as in Equation 2:

2 $${f}_{(ps-max)}=(\frac{ur{d}_{(psp)}}{ur{d}_{(psp)}+1})$$

By inserting Equation 2 into equation 1, the maximum probability *P*_(*max*)_ of assigning the allele found in MCE1356 to the wrong parental species was calculated as in Equation 3:

3 $${P}_{(max)}={(\frac{ur{d}_{(psp)}}{ur{d}_{(psp)}+1})}^{ur{d}_{(psp)}}\times (1-(\,\frac{ur{d}_{(psp)}}{ur{d}_{(psp)}+1}))$$

Results revealed that with a unique read depth of two, three and four in each parental species, the maximum probability of assigning the allele found in MCE1356 to the wrong parental species was 0.148, 0.105 and 0.082, respectively. These maximum values should not be interpreted as error rates, as they would only be obtained if all sites were variable within the parental species, and all sites had a MAF of exactly (1 − (urd/urd + 1)). Furthermore, assuming that the MAF distributions in belugas and narwhals were similar, erroneous assignment of alleles found in MCE1356 would affect both species equally, and therefore have a minimal influence on inferences of hybridization. A benefit of using the unique read depth in equations 2 and 3 was that it combined the number of individuals and number of reads in the estimation of the probability of assigning the allele found in MCE1356 to the wrong parental species. Subsequently, the read depth distribution, MAF (with fixed major and minor allele), and number of individuals with data in each site were calculated separately for each parental species.

Three datasets were constructed, which besides MCE1356 included parental species panels with minimum unique read depths of two, three and four. The number of sites retained in the three datasets was 61,105, 11,764 and 360, respectively. As a compromise between maximizing the number of sites and minimizing the wrong assignment of alleles found in MCE1356, we chose to use the dataset with one read in MCE1356 and minimum unique read depths of three in each parental species. That dataset included 11,764 sites, which were used in subsequent analyses.

Summary statistics were performed on the dataset with 17 individuals, including number of sites that were (i) fixed for different alleles in the beluga and narwhal species panels; (ii) polymorphic in belugas, but not in narwhals; (iii) polymorphic in narwhals, but not in belugas; (iv) polymorphic in both belugas and narwhals. The sites that are estimated to be fixed between the two parental species panels will be enriched for markers that are highly differentiated, i.e. have large allele frequency differences, between the two parental species. These markers, although not necessarily fixed differences between the two parental species, are still highly informative for ancestry in MCE1356.

We used the genotype likelihoods from the dataset without MCE1356 to verify that the belugas and narwhals in the reference panel were not themselves recently admixed individuals. We estimated their individual admixture coefficients while specifying two populations (K = 2) using NgsAdmix^28^. One hundred runs were performed and mean and standard deviation of the admixture coefficients were used for subsequent interpretation. To confirm that the filters had not revealed previously hidden admixed genetic profiles in the reference panel, this analysis was performed both before and after the unique read depth filters were applied.

We analysed the admixture proportions of MCE1356 using fastNGSadmix^29^, applying 1,000 bootstraps. fastNGSadmix uses allele frequencies of reference populations/species and the genotype likelihoods of a single individual to estimate its admixture proportions. The software has proven robust with NGS data with coverage as low as 0.00015x^29^, and was therefore ideal for our study.

We further estimated the hybrid status of MCE1356 by investigating sites fixed for different alleles in the beluga panel and the narwhal panel (9,178 sites), and comparing the observed proportion of reads matching the beluga-specific allele and the narwhal-specific allele in MCE1356 to the expected proportions under different hybridization scenarios. To determine how well seven different hybridization scenarios matched the observed data, we computed a Pearson’s Chi-square goodness of fit statistic. The test statistic is computed as in Equation 4:

4 $$T=\Sigma (\frac{{(Oi-Ei)}^{2}}{Ei})$$

where O_i_ and E_i_ are the observed and expected counts of alleles derived from parental species i, respectively. Under the null hypothesis where the chosen scenario corresponds well to the observed data, the test statistic T follows a central χ^2^ distribution. Thus, the scenario that corresponds best with the observed data would lead to the lowest test statistic.

To further investigate the seven different hybridization scenarios, we computed the likelihood of the observed alleles at sites that were fixed for different alleles in the parental populations. Specifically, we computed the likelihood of the observed alleles in the hybrid MCE1356, under a binomial model for the inheritance of the alleles from the parental species, further assuming independence between markers. We would like to note, however, that the violation of the independence assumption still leads to unbiased estimates. Assuming that the hybrid MCE1356 is composed of a proportion *b* of beluga ancestry and (1-b) of narwhal ancestry, we can write the likelihood of *b* as a product of the likelihood at *k* independent sites, given the number of reads n_*ib*_ that match the beluga allele at site i and n_*in*_ that match narwhal ancestry, as in Equation 5.

5 $$L(b|{n}_{b},{n}_{n})={\prod }_{i=1}^{k}L(b|{n}_{ib},{n}_{in})={\prod }_{i=1}^{k}P({n}_{ib},{n}_{in}|b)={\prod }_{i=1}^{k}(\begin{array}{c}{n}_{ib}+{n}_{in}\\ {n}_{ib}\end{array}){b}^{{n}_{ib}}{(1-b)}^{{n}_{in}}$$

We computed the log-likelihood of *b* across its whole range, from 0 to 1, and compared the log-likelihoods across the seven different hybridization scenarios.

### Sex determination

To determine the sex of MCE1356 and the individuals in the beluga and narwhal reference panels, we investigated X chromosome to autosomal coverage ratios. This was done by comparing the coverage across scaffolds putatively originating from the X chromosome, to that of scaffolds putatively originating from the autosomes. We determined which scaffolds originate from the X chromosome by aligning the killer whale genome to the Cow (*Bos taurus*) X chromosome (CM008168.2) with SatsumaSynteny^30^. Moreover, to remove biases that may occur due to homology between some regions of the X and Y chromosomes, we further aligned the resultant putative X chromosome scaffolds to the human Y chromosome (NC_000024.10) and removed these scaffolds from further analysis. We then calculated coverage across the remaining X scaffolds and the four largest scaffolds not aligning to the X chromosome (i.e. autosomal scaffolds), using the depth function in SAMtools^17^. To compensate for any variation in coverage across the genome, we randomly selected 10,000,000 sites, calculated the average coverage for these sites, and repeated this step 100 times. For each individual, 5% and 95% confidence intervals, as well as first and third quartiles, were calculated and used for subsequent interpretation.

### Stable carbon and nitrogen isotope analysis

Powdered bone samples (~100 mg) from MCE1356, 18 beluga and 18 narwhal skulls were treated with 10 ml 2:1 chloroform/methanol (v/v) under sonication for 1 h to remove lipids. After removing the solvent, the samples were dried under normal atmospheric pressure for 24 h. Samples were then demineralized in 10 ml of 0.5 M HCl for 4 h while being agitated by an orbital shaker. After demineralization, samples were rinsed to neutrality with Type I water, and then heated at 75 °C for 36 h in 10^−3^ M HCl to solubilize the collagen. The water soluble collagen was then freeze dried. The collagen extract was then treated with 10 ml 10:5:4 chloroform/methanol/water (v/v/v) under sonication for 1 h to remove any residual lipids. After centrifugation, the chloroform/methanol layer was removed and the methanol was evaporated out of the water layer at 60 °C for 24 h. The samples were again freeze dried and weighed into tin capsules for elemental and isotopic analysis. Carbon and nitrogen stable isotopic and elemental compositions were determined using a Nu Horizon (Nu Instruments, UK) continuous flow isotope ratio mass spectrometer at Trent University, Canada. Stable carbon and nitrogen isotopic compositions were calibrated relative to the VPDB and AIR scales using USGS40 and USGS41a. Standard uncertainty was determined to be ±0.17‰ for *δ*^13^C and ±0.22‰ for *δ*^15^N^31^; additional analytical details are provided in Supplementary Document S4. The statistical significance of differences between beluga and narwhal isotopic compositions were assessed using unpaired t-tests.

## Results

### Mitochondrial analysis

**Figure 3:**
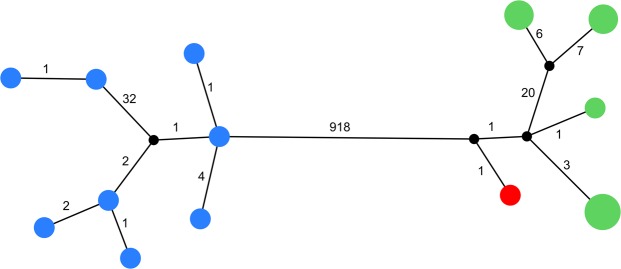
Median-spanning haplotype network of complete mitochondrial genomes of eight belugas (blue), eight narwhals (green) and MCE1356 (red). Black dots indicate intermediate haplotypes not found in the data. The size of the circles indicate the relative number of specimens sharing a haplotype. Numbers indicate number of variable sites between haplotypes.

Mapping the reads of MCE1356 to the narwhal mitochondrial reference genome yielded a ~3-fold higher coverage (19.5x) than when reads were mapped to the beluga reference genome (6.6x), indicating a narwhal maternal lineage (Supplementary Table S5). We retrieved an average coverage of the mitochondrial genomes of the beluga and narwhal reference panel of 69x and 84x, respectively (Supplementary Table S5). MCE1356 grouped with the narwhal clade both when it was mapped to the beluga (Supplementary Fig. S6) and narwhal reference genome (Fig. 3), confirming a narwhal maternal lineage.

### Nuclear analysis

The beluga and narwhal tissue samples were sequenced to an average coverage of 0.24x and 0.18x, respectively, and the sequencing of the four MCE1356 libraries yielded a combined coverage of 0.05x (Supplementary Table S5). The read depth distribution of the combined dataset, excluding reads below 25 bp, duplicate reads, reads with Phred score below 30, mapping quality (MAPQ from BWA) below 25, reads mapping to multiple locations in the reference genome, but including sites covered only by a single read and non-variable sites, had a mean read depth of 4.14x (Supplementary Fig. S7). After removing sites that were not variable with a *p* value below 1e^−4^, and sites with a read depth greater than nine, the dataset including 17 samples contained 2,700,875 polymorphic sites and the dataset without MCE1356 included 2,671,704 sites.

Excluding sites with no reads in MCE1356 reduced the number of sites to 107,997, with 105,588 sites covered by one read in MCE1356, 2,325 sites covered by two reads and 84 sites covered by three or more reads. Filtering to only include nuclear sites with (i) one read in MCE1356, (ii) minimum beluga unique read depth of three, (iii) minimum narwhal unique read depth of three, resulted in a final dataset including 11,764 SNPs, of which 9,178 sites (78.0%) were fixed for alternate alleles in belugas and narwhals; 1,553 sites (13.2%) were polymorphic in belugas, but not in narwhals; 724 sites (6.2%) were polymorphic in narwhals, but not in belugas; 309 sites (2.6%) were polymorphic in both belugas and narwhals. The mean read depth in the final dataset was 8.15x and the mean number of belugas and narwhals per SNP in the reference panels was 3.34 (SD = 0.56) and 3.26 (SD = 0.49), respectively.

In the NGSAdmix analyses performed on 2,671,704 sites, all belugas and narwhals had mean admixture coefficients >0.999 (SD <10^−6^) indicating that none of the individuals in the reference panel were recently admixed. This result was retained when applying the filters based on read depth in the reference panel, reducing the number of sites to 11,764.

**Figure 4:**
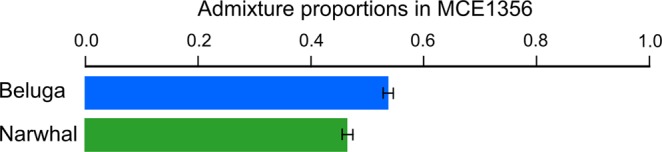
Admixture proportions of MCE1356 from fastNGSadmix. Error bars are standard deviations estimated from 1000 bootstraps.

The FastNGSadmix analysis that estimated the admixture proportions in MCE1356 from its genotype likelihoods and allele frequencies of belugas and narwhals across 11,764 sites, estimated that MCE1356 is 54% beluga and 46% narwhal (Fig. 4), and indicating that it is a first generation hybrid.

**Figure 5:**
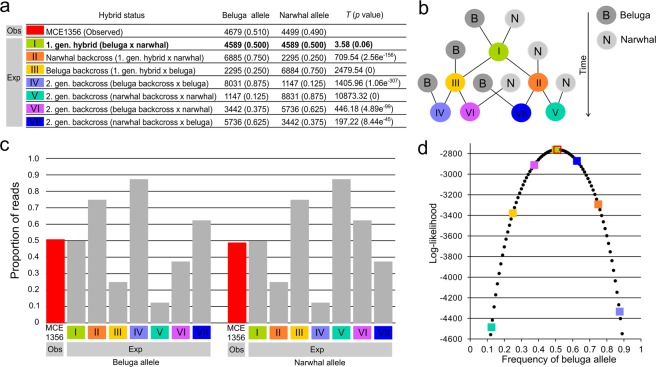
Distribution of reads in MCE1356 at 9,178 polymorphic sites fixed for altering alleles in reference panel belugas and narwhals. (**a**) Observed number of MCE1356 reads matching the beluga allele and narwhal allele, and expected number of reads matching the beluga allele and narwhal allele under seven hybridization scenarios (I–VII). *T* values from Pearson’s Chi-square goodness of fit statistic, where MCE1356 is the observed and the seven different hybridization scenarios are used to compute the expected read counts. The only hybridization scenario that could not be rejected is presented in bold. (**b**) Schematic illustration of the seven hybridization scenarios. (**c**) Observed proportion of reads matching the beluga allele and the narwhal allele in MCE1356 and the expected proportions matching the beluga allele and the narwhal allele under seven different hybridization scenarios. (**d**) Log-likelihood of *b*, the proportion of beluga ancestry in MCE1356, across its whole range (see Equation 5 in main text) and the log-likelihoods of the seven different hybridization scenarios. The seven hybrid scenarios are represented by colored squares with colors matching 5a, MCE1356 is represented in red.

Among the 9,178 sites that were called fixed between the beluga and narwhal species panels and covered by a single read in MCE1356, 4,679 sites (51%) carried the beluga-specific allele and 4,499 sites (49%) had the narwhal-specific allele (Fig. 5a). When comparing this observed read distribution with the expected read distribution under seven different hybridization scenarios (Fig. 5b), the *T* statistics and the associated *p* values revealed that only the first-generation-hybrid scenario could not be rejected (*T* value = 3.58, p value = 0.06) (Fig. 5a,c). The log-likelihood of the proportion of beluga/narwhal ancestry, given the observed read counts, under the seven hybridization scenarios further supported that MCE1356 was a first generation hybrid (Fig. 5d).

### Sex determination

**Figure 6:**
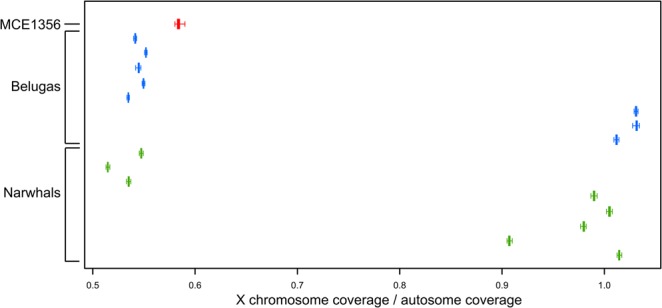
X chromosome to autosome coverage ratio in MCE1356 and the eight belugas and eight narwhals in the reference panel. Values close to 0.5 indicate the individual is a male. Values close to1 indicate a female.

We used the X chromosome to autosome coverage ratios to determine the sex of MCE1356. A 1:2 ratio (0.5) indicates a single copy of the X chromosome and a male individual, and a 1:1 ratio (1) indicates two copies of the X chromosome and a female individual. We find a ratio of ~0.58 in MCE1356, suggesting the specimen was likely male. To investigate this ratio further, we performed the same analysis on the 16 individuals of the reference panel (Fig. 6). We found no individuals to have a perfect 1:2 (0.5) X chromosome to autosome coverage ratio. This discrepancy could arise due to a multitude of factors, including random chance, sequencing bias, and difficulties stemming from the genome assembly and correct X chromosome scaffold determination. However, it was obvious that there were clear differences between a number of individuals, and these differences could be separated into two clusters (sexes) around ~0.5 and ~1, with very little variation within each individual. These results gave us confidence in our method. In the reference panel, we found eight males and eight females. Furthermore, despite the much lower average coverage of MCE1356, the X chromosome to autosome coverage ratio of ~0.58, with little variation between different subsamples, still clearly clustered with the putative males from the reference panel.

### Stable isotope analysis

**Figure 7:**
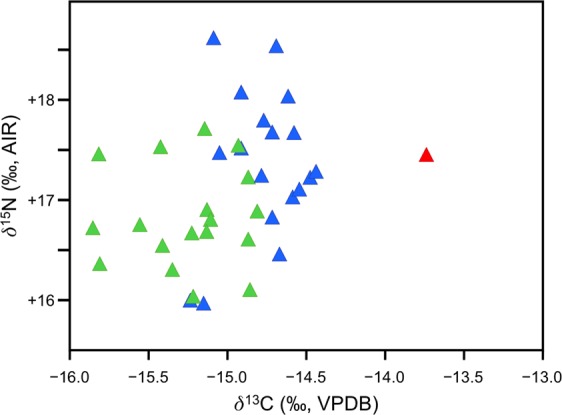
Stable carbon and nitrogen isotopic compositions of belugas (blue), narwhals (green) and MCE1356 (red).

The carbon and nitrogen isotopic compositions of beluga, narwhal, and MCE1356 are summarized in Fig. 7 and presented in full in the Supplementary Information, along with elemental compositions and collagen yields (Supplementary Document S4). Belugas and narwhals were characterized by significantly different *δ*^13^C (*p* < 0.001) and *δ*^15^N (*p* = 0.02) values. MCE1356 had a much higher *δ*^13^C value than any of the belugas or narwhals analyzed (>3σ higher than the beluga mean; >4σ higher than the narwhal mean), but a comparable *δ*^15^N value.

## Discussion

Our analyses of genome-wide DNA retrieved from MCE1356 and a reference panel of belugas and narwhals showed that the abnormal skull is an F1 hybrid of the two species (Figs 4, 5). Mating strategies of belugas and narwhals are not well understood, reflecting the logistical challenges associated with studying the behavior of Arctic marine endemics; mating takes place in spring, when the whales are particularly difficult to access due to sea ice break up. However, the existence of a beluga/narwhal hybrid indicates that the two species can mate and produce viable offspring.

Our analysis of the mitochondrial genome shows that the mother of MCE1356 was a narwhal (Fig. 3). Male narwhals have one, and in rare cases two, spiraled tusks representing a protruding canine tooth. In contrast, belugas have up to 40 homodont teeth (Fig. 2). The narwhal tusk has been hypothesized to be a secondary sexual characteristic of the species^32^, which could suggest that male belugas would be less successful in securing cross-species matings than male narwhals. In addition, recent work has suggested that belugas and narwhals have different mating systems, with sperm competition being more important in belugas than in narwhals^33^. Our finding of a narwhal mother and beluga father of MCE1356 suggests that even with the absence of a tusk and different mating systems, successful mating can still occur between a male beluga and a female narwhal.

The elongated mating period (late winter to late spring) and migratory nature of both species makes it difficult to evaluate the overlap in their respective distributions during the mating season. However, Disko Bay in West Greenland, where the hybrid skull was collected (Fig. 1c), is one of only a few areas globally where belugas and narwhals are known to occur in large and predictable numbers during the mating season^7^. Hybridization could also occur in mixed species aggregations, as belugas are occasionally found in narwhal pods^7^, and narwhals have also been continuously observed in beluga pods.

The admixture proportions inferred from 11,764 variable sites indicated that MCE1356 was a first-generation beluga/narwhal hybrid. This was supported by our analyses of 9,178 sites fixed for alternative alleles in the two parental species, where the observed distribution of reads matched the expected distribution in a first-generation hybrid (Fig. 5a,c). The coverage of the X chromosome was roughly half of the autosome, revealing that MCE1356 was a male (Fig. 6). Considering the large size of the MCE1356 skull^8^, it seems credible, as both beluga and narwhal males are larger than females^1,2^.

Our finding of hybridisation between belugas and narwhals is unexpected, as a recent genomic analysis showed that gene flow between the two species ceased 1.25-1.65 MYA^4^. However, hybridization among cetacean species is relatively common^34^; there are at least 16 described cases of hybridization between wild or captive cetacean species^35^. Evidence has been based on morphological traits in the offspring and genetic analyses^36–38^. A recent genomic study of eight rorqual species (baleen whales) suggested pronounced hybridization throughout the evolution and speciation of the group^39^.

The differences between the carbon and nitrogen isotopic compositions in belugas and narwhals, while relatively small, suggest distinct foraging behaviors, consistent with what has been reported for these two species in the North Water Polynya^40^. The unusually high *δ*^13^C value for MCE1356 suggests a unique diet for this individual. Because bone collagen remodels at a slow rate, reflecting the diet over the last several years of an animal’s life^41^, this unique diet reflects sustained differences in prey consumption or habitat use, and cannot be ascribed to unusual short-term variation. High *δ*^13^C values are observed in marine animals that forage to a greater extent on benthic relative to pelagic prey^42^. Accounting for the differences in fractionation with trophic level, bearded seals (*Erignathus barbatus*) and walrus (*Odobenus rosmarus*) tend to have the highest *δ*^13^C values of any Arctic marine mammal, consistent with their strong reliance on benthic prey^40^.

The high *δ*^13^C value of the hybrid MCE1356 therefore suggests a greater use of benthic relative to pelagic prey compared to either belugas or narwhals foraging in the same area, the magnitude of which may be comparable to the difference in benthic prey consumption between bearded (*Erignathus barbatus*) and ringed (*Pusa hispida*) seals, which is substantial^43^. The unique foraging behavior of the beluga/narwhal hybrid may have been driven by its peculiar dental morphology (Fig. 2b, Supplementary Fig. S1). Although the precise prey species that formed part of its diet whilst alive are difficult to discern, the *δ*^15^N value suggests that the mean trophic level of prey consumed would have been comparable to belugas and narwhals, for which fish and squid are important prey items^10,44,45^.

## Conclusions

Recent major advances in analytical methods that use genotype likelihoods, which take uncertainty in genotype calling into account rather than using directly called genotypes, are revolutionizing the quality of insights gained from low-coverage genomic data. Even though we had an average coverage of less than 30% of the genome per individual in the beluga and narwhal reference panel, and only 5% of the hybrid genome, we were still able to retain 11,764 informative sites. From these sites, we confirm hybridization between belugas and narwhals based on DNA retrieved from an anomalous skull. We analyzed 9,178 sites fixed for alternative alleles in each parental species and ascertain that specimen MCE1356 is an F1 hybrid, used mitochondrial genomes to show the mother was a narwhal, and investigated the coverage of scaffolds mapping to the X chromosome versus those of the autosomes to infer the hybrid was male. In addition, the stable isotopic signature of carbon and nitrogen indicates a unique dietary niche of the hybrid unlike that of either parental species, supported by its unique dentition.

## Supplementary information

Hybridization between Arctic cetaceans Supplementary

## Data Availability

Data is available from Electronic Research Data Archive: http://www.erda.dk/public/archives/YXJjaGl2ZS16NXRqTXE=/published-archive.html.
