# Supplementary material for: Hybridization between two high Arctic cetaceans confirmed by genomic analysis
Source: Sci Rep. 2019 Jun 20;9:7729. doi: 10.1038/s41598-019-44038-0 (PMC6586676; doi:10.1038/s41598-019-44038-0)

Hybridization between two high Arctic cetaceans confirmed by genomic analysis

Authors

Mikkel Skovrind, Jose Alfredo Samaniego Castruita, James Haile, Eve C. Treadaway, Shyam Gopalakrishnan, Michael V. Westbury, Mads Peter Heide Jørgensen, Paul Szpak, Eline D. Lorenzen

Supplementary Figure S1

Teeth from MCE1356 from which DNA was retrieved. The skull and teeth are housed at the Natural History Museum of Denmark, University of Copenhagen


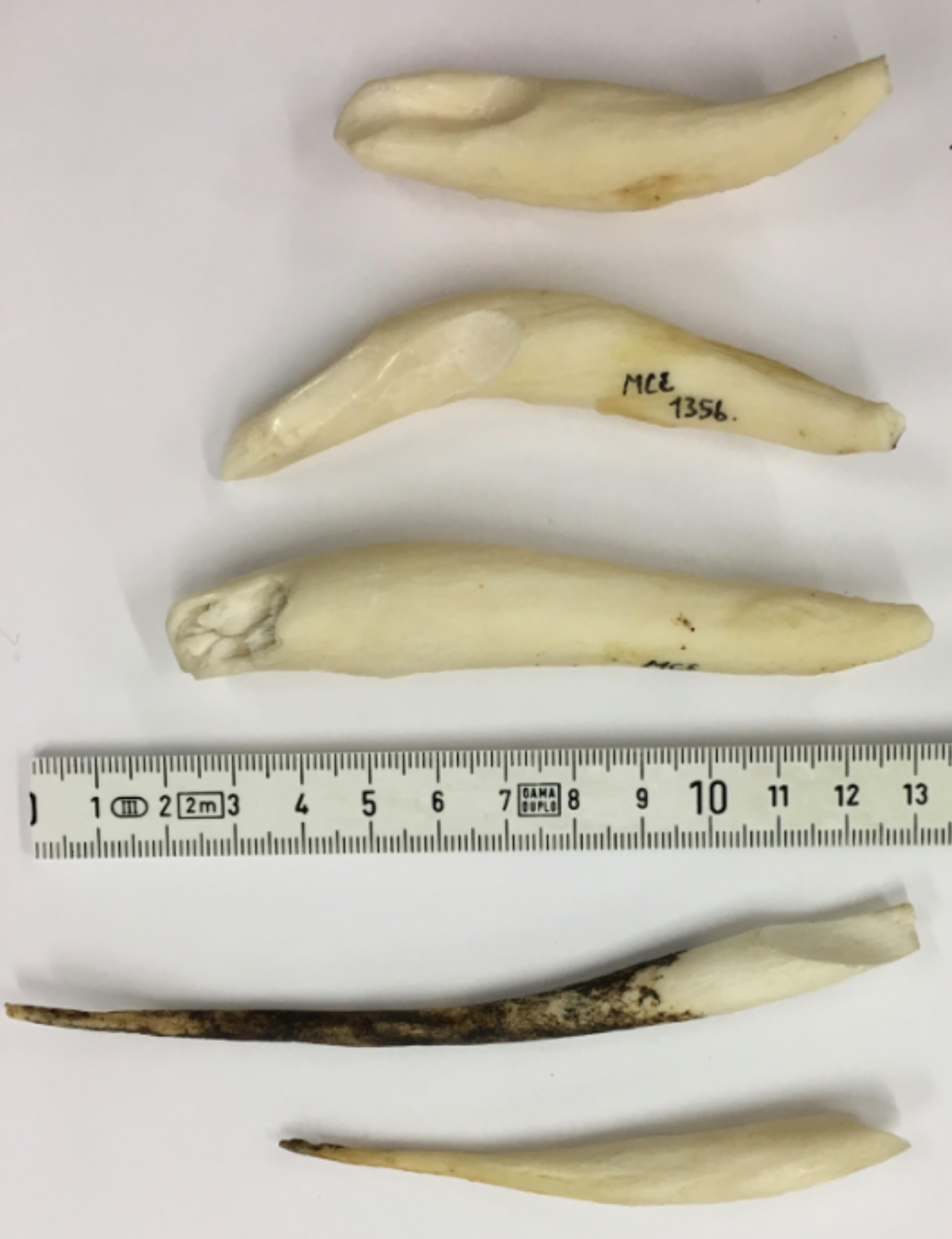
Supplementary Table S2

Sample information. All samples are housed at the Natural History Museum of Denmark, University of Copenhagen.

Supplementary Figure S3

MapDamage plot of base frequencies and substitutions in DNA sequence reads from MCE1356. (a) Base frequencies inside (surrounded by grey outline) and outside the reads. (b) Substitutions from the 5’ (left) and the 3’ end (right). C -> T substitutions (red), G -> A substitutions (blue), insertions relative to the killer whale reference (purple), and all other substitutions (grey).


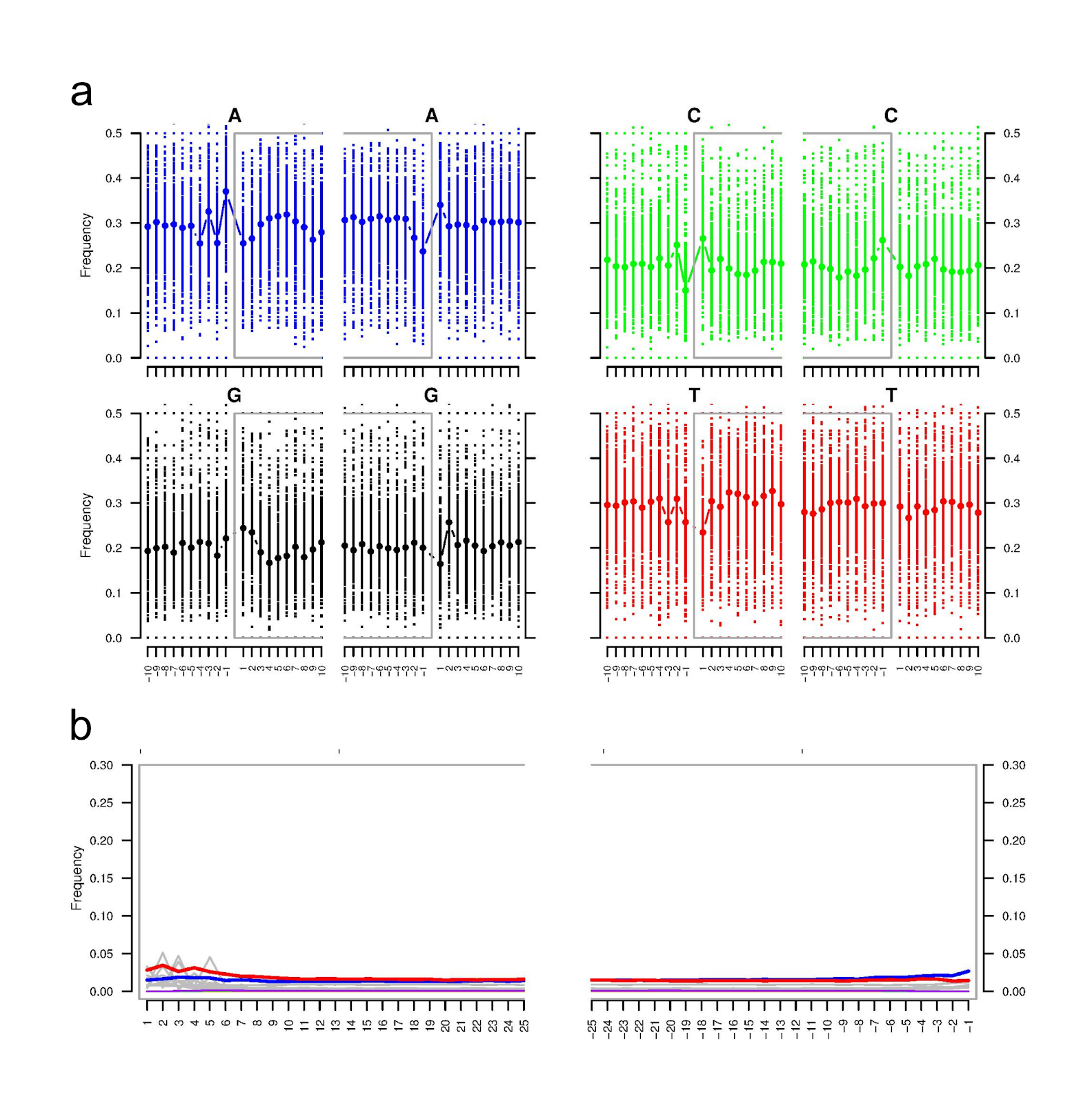


Supplementary Document S4

**Supplementary Material**

**Stable Isotope Analysis − Calibration, Accuracy, and Precision**

Carbon and nitrogen isotopic and elemental compositions were determined using a Nu Horizon continuous flow isotope ratio mass spectrometer. Sample measurements were calibrated relative to VPDB (*δ*^13^C) and AIR (*δ*^15^N) using USGS40 and USGS41a (Table S1; Qi et al., 2003; Qi et al., 2016).

**Table S1.** Standard reference materials used for calibration of *δ*^13^C relative to VPDB and *δ*^15^N relative to AIR.

| Standard | Material | Accepted *δ*^13^C  (‰, VPDB) | Accepted *δ*^15^N  (‰, AIR) |
| --- | --- | --- | --- |
| USGS40 | Glutamic Acid | −26.39±0.04 | −4.52±0.06 |
| USGS41a | Glutamic Acid | +36.55±0.08 | +47.55±0.15 |

The following standards were used to monitor accuracy and precision (Table S2). The isotopic compositions for the internal keratin standard represent long-term averages.

**Table S2.** Standard reference materials used to monitor internal accuracy and precision.

| Standard | Material | Mean *δ*^13^C  (‰, VPDB) | Mean *δ*^15^N  (‰, AIR) |
| --- | --- | --- | --- |
| SRM−1 | Caribou bone collagen | −19.36±0.11 | +1.81±0.10 |
| SRM−2 | Walrus bone collagen | −14.76±0.12 | +15.59±0.11 |
| SRM−4 | Gluten | −26.76±0.08 | +5.25±0.11 |
| SRM−14 | Polar bear bone collagen | −13.66±0.06 | +21.63±0.11 |

Table S3 summarizes the mean and standard deviation of carbon and nitrogen isotopic compositions for all check standards, as well as the standard deviation for all calibration standards – the mean of the calibration standard for an individual run is predetermined to calibrate the data.

Table S3. Mean and standard deviation of all check and calibration standards for all analytical sessions containing data presented in this paper. Note that means for calibration standards are not presented as they are pre-determined to be equal to the known value.

| **Run ID** | **Standard** | **n** | ***δ*^13^C (‰, VPDB)** | | | ***δ*^15^N (‰, AIR)** | | |
| --- | --- | --- | --- | --- | --- | --- | --- | --- |
| 18-02 | USGS40 | 5 |  | ± | 0.01 |  | ± | 0.25 |
| 18-03 | USGS40 | 5 |  | ± | 0.13 |  | ± | 0.06 |
| 18-06 | USGS40 | 8 |  | ± | 0.11 |  | ± | 0.06 |
| 18-02 | USGS41a | 5 |  | ± | 0.05 |  | ± | 0.28 |
| 18-03 | USGS41a | 5 |  | ± | 0.14 |  | ± | 0.06 |
| 18-06 | USGS41a | 8 |  | ± | 0.05 |  | ± | 0.03 |
| 18-06 | SRM−1 | 3 | −19.39 | ± | 0.02 | +1.72 | ± | 0.06 |
| 18-02 | SRM−2 | 4 | −14.86 | ± | 0.07 | +15.79 | ± | 0.12 |
| 18-03 | SRM−2 | 4 | −14.84 | ± | 0.08 | +15.62 | ± | 0.07 |
| 18-06 | SRM−2 | 6 | −14.78 | ± | 0.03 | +15.50 | ± | 0.03 |
| 18-02 | SRM−4 | 6 | −26.76 | ± | 0.02 | +5.19 | ± | 0.11 |
| 18-03 | SRM−4 | 5 | −26.72 | ± | 0.12 | +5.28 | ± | 0.04 |
| 18-02 | SRM−14 | 4 | −13.65 | ± | 0.04 | +21.78 | ± | 0.07 |
| 18-03 | SRM−14 | 4 | −13.70 | ± | 0.09 | +21.59 | ± | 0.11 |
| 18-06 | SRM−14 | 6 | −13.71 | ± | 0.03 | +21.41 | ± | 0.05 |

Twenty-eight samples were analyzed in duplicate. The pooled standard deviation for the duplicate pairs was ±0.15 ‰ for *δ*^13^C and 0.12 ‰ for *δ*^15^N.

**Analytical Uncertainty**

Standard uncertainty was calculated using the method present by Szpak et al. (2017b), which largely follows Magnusson et al.’s (2012) approach. Standard uncertainty was determined to be ±0.17 ‰ for *δ*^13^C and ±0.22 ‰ for *δ*^15^N.

**Results**

The carbon and nitrogen isotopic and elemental compositions of all samples analyzed are presented in Table S4. Most of the samples were analyzed in duplicate and the results of each analysis is presented separately in Table S4. Four samples that were analyzed were excluded from subsequent analysis because they presented unusually high atomic C:N ratios and are identified with strikethrough text in Table S4. The C:N ratios of archaeological specimens are used to assess the integrity of the collagen as contaminated or degraded collagen will tend to have isotopic compositions that are significantly different from the original, endogenous material (Ambrose, 1990; DeNiro, 1985; Szpak et al., 2017a; van Klinken and Hedges, 1995). For modern material, collagen degradation is no an issue but contamination with lipids is a potential concern as the *δ*^13^C values of lipids are significantly lower than those of proteins such as collagen (Post et al., 2007). Even though these samples were treated with solvents that are effective at removing lipids from bone (Guiry et al., 2016), some still had atomic C:N ratios that suggested contamination with additional material that was rich in carbon, lacking in nitrogen, and had a low *δ*^13^C value – most likely lipids. The samples that were excluded had atomic C:N ratios in the range of 3.56 to 3.92, whereas the theoretical value for pure mammalian bone collagen is 3.23 (Szpak, 2011). These samples also displayed the lowest *δ*^13^C values of any members of the taxon to which they belonged. Consequently the isotopic compositions were considered unreliable and were excluded from plots and statistical calculations.

Table S4. Stable isotopic and elemental compositions for all samples analyzed in this study.

| Lab ID | Sample ID (MCE) | Location | Year | Taxon | *δ*^13^C (‰) | *δ*^15^N (‰) | wt%C | wt%N | Atomic C:N | *δ*^13^C (‰) | *δ*^15^N (‰) | wt%C | wt%N | Atomic C:N |
| --- | --- | --- | --- | --- | --- | --- | --- | --- | --- | --- | --- | --- | --- | --- |
| 571 | 1312 | Kullorsuaq | 1990 | Delphinapterus leucas | -15.04 | 17.46 | 49.30 | 16.90 | 3.40 |  |  |  |  |  |
| 572 | 1313 | Kullorsuaq | 1990 | Delphinapterus leucas | -14.77 | 17.19 | 36.40 | 12.90 | 3.29 | -14.76 | 17.25 | 35.0 | 12.4 | 3.29 |
| 573 | 1314 | Kullorsuaq | 1991 | Delphinapterus leucas | -14.47 | 17.00 | 34.03 | 12.58 | 3.15 | -14.56 | 17.19 | 34.7 | 12.7 | 3.19 |
| 587 | 1316 | Nuusuaaq | 1994 | Delphinapterus leucas | -14.68 | 18.44 | 44.45 | 15.38 | 3.37 |  |  |  |  |  |
| 588 | 1318 | Nuusuaaq | 1994 | Delphinapterus leucas | -15.16 | 15.97 | 46.09 | 15.77 | 3.41 | -15.12 | 15.93 | 44.8 | 15.5 | 3.37 |
| 589 | 1319 | Nuusuaaq | 1994 | Delphinapterus leucas | -14.72 | 18.07 | 44.84 | 15.70 | 3.33 | -15.09 | 18.07 | 46.3 | 15.8 | 3.42 |
| 590 | 1320 | Nuusuaaq | 1994 | Delphinapterus leucas | -14.41 | 17.87 | 43.77 | 15.50 | 3.29 | -14.78 | 18.20 | 46.0 | 15.9 | 3.37 |
| 591 | 1321 | Nuusuaaq | 1994 | Delphinapterus leucas | -15.07 | 18.61 | 44.97 | 15.29 | 3.43 |  |  |  |  |  |
| 592 | 1322 | Nuusuaaq | 1994 | Delphinapterus leucas | -15.24 | 15.97 | 44.98 | 15.03 | 3.49 | -15.21 | 16.01 | 45.4 | 15.0 | 3.53 |
| 593 | 1323 | Nuusuaaq | 1994 | Delphinapterus leucas | -14.35 | 17.12 | 43.29 | 15.79 | 3.20 | -14.50 | 17.39 | 44.2 | 15.9 | 3.24 |
| 594 | 1324 | Nuusuaaq | 1994 | Delphinapterus leucas | -14.37 | 17.59 | 43.43 | 16.01 | 3.16 | -14.76 | 17.73 | 44.7 | 16.0 | 3.26 |
| 595 | 1325 | Nuusuaaq | 1994 | Delphinapterus leucas | -14.38 | 17.17 | 44.78 | 16.16 | 3.23 | -14.52 | 17.27 | 45.3 | 16.1 | 3.28 |
| 596 | 1326 | Nuusuaaq | 1994 | Delphinapterus leucas | -14.77 | 17.34 | 44.75 | 15.82 | 3.30 | -15.06 | 17.68 | 45.9 | 16.0 | 3.35 |
| 599 | 1329 | Nuusuaaq | 1994 | Delphinapterus leucas | -14.71 | 17.67 | 45.08 | 15.76 | 3.34 |  |  |  |  |  |
| 600 | 1330 | Nuusuaaq | 1994 | Delphinapterus leucas | -14.71 | 16.79 | 44.32 | 15.56 | 3.32 | -14.69 | 16.85 | 44.1 | 15.9 | 3.24 |
| 601 | 1331 | Nuusuaaq | 1994 | Delphinapterus leucas | -14.68 | 17.72 | 44.93 | 16.46 | 3.18 | -14.83 | 17.81 | 43.4 | 15.6 | 3.24 |
| 602 | 1332 | Nuusuaaq | 1994 | Delphinapterus leucas | -14.51 | 16.35 | 43.65 | 15.92 | 3.20 | -14.81 | 16.55 | 43.9 | 15.3 | 3.35 |
| 603 | 1333 | Nuusuaaq | 1994 | Delphinapterus leucas | -14.53 | 16.99 | 44.58 | 16.18 | 3.21 | -14.61 | 17.06 | 43.3 | 15.5 | 3.26 |
| 575 | 1356 | Kitsissuarsuit | 1986 | Delphinapterus leucas X Monodon monoceros | -13.78 | 17.40 | 44.84 | 16.33 | 3.20 | -13.91 | 17.64 | 44.8 | 16.5 | 3.17 |
| 576 | 1358 | Uummannaq | 1993 | Monodon monoceros | -14.77 | 17.17 | 44.27 | 15.83 | 3.26 | -14.97 | 17.31 | 45.8 | 16.3 | 3.28 |
| 577 | 1359 | Uummannaq | 1993 | Monodon monoceros | -15.83 | 17.48 | 45.5 | 15.3 | 3.47 |  |  |  |  |  |
| 580 | 1360 | Uummannaq | 1993 | Monodon monoceros | -15.15 | 17.72 | 44.04 | 15.53 | 3.31 |  |  |  |  |  |
| 581 | 1361 | Uummannaq | 1993 | Monodon monoceros | -15.07 | 16.79 | 45.45 | 16.49 | 3.21 | -15.21 | 17.04 | 44.7 | 16.1 | 3.24 |
| 582 | 1362 | Uummannaq | 1993 | Monodon monoceros | -15.14 | 16.68 | 42.78 | 15.58 | 3.20 | -15.34 | 16.70 | 44.4 | 15.8 | 3.28 |
| 583 | 1363 | Uummannaq | 1993 | Monodon monoceros | -15.40 | 16.62 | 44.30 | 15.74 | 3.28 | -15.72 | 16.91 | 45.8 | 15.8 | 3.38 |
| 584 | 1364 | Balgoni | 1993 | Monodon monoceros | -15.81 | 16.38 | 42.84 | 15.04 | 3.32 |  |  |  |  |  |
| 585 | 1365 | Balgoni | 1993 | Monodon monoceros | -15.34 | 17.38 | 44.79 | 15.94 | 3.28 | -15.51 | 17.70 | 44.7 | 15.9 | 3.28 |
| 586 | 1366 | Balgoni | 1993 | Monodon monoceros | -14.82 | 17.50 | 43.65 | 15.75 | 3.23 | -15.05 | 17.61 | 45.2 | 16.1 | 3.27 |
| 605 | 1380 | Qaanaaq | 2003 | Monodon monoceros | -15.42 | 16.55 | 42.71 | 15.04 | 3.31 |  |  |  |  |  |
| 607 | 1382 | Qaanaaq | 2003 | Monodon monoceros | -15.14 | 16.68 | 44.56 | 15.54 | 3.34 | -15.13 | 16.71 | 44.9 | 15.7 | 3.33 |
| 608 | 1383 | Qaanaaq | 2003 | Monodon monoceros | -15.22 | 16.05 | 44.68 | 15.87 | 3.28 |  |  |  |  |  |
| 609 | 1384 | Qaanaaq | 2003 | Monodon monoceros | -14.82 | 16.90 | 43.93 | 16.16 | 3.17 |  |  |  |  |  |
| 610 | 1386 | Qaanaaq | 2003 | Monodon monoceros | -15.03 | 16.79 | 43.46 | 15.95 | 3.18 | -15.20 | 16.84 | 43.9 | 15.7 | 3.26 |
| 611 | 1388 | Qaanaaq | 2003 | Monodon monoceros | -14.92 | 16.65 | 42.02 | 15.67 | 3.13 | -14.83 | 16.61 | 43.2 | 15.7 | 3.21 |
| 612 | 1389 | Qaanaaq | 2003 | Monodon monoceros | -14.80 | 16.14 | 44.72 | 16.48 | 3.16 | -14.92 | 16.12 | 43.3 | 15.8 | 3.20 |
| 613 | 1374 | Qaanaaq | 2003 | Monodon monoceros | -15.11 | 16.24 | 42.84 | 15.49 | 3.22 | -15.60 | 16.41 | 45.4 | 15.4 | 3.44 |
| 615 | 1377 | Qaanaaq | 2003 | Monodon monoceros | -15.86 | 16.73 | 44.61 | 16.12 | 3.23 |  |  |  |  |  |

**References**

Ambrose, S. H., 1990, Preparation and characterization of bone and tooth collagen for isotopic analysis, *Journal of Archaeological Science*, **17**(**4**), 431-451.

DeNiro, M. J., 1985, Postmortem preservation and alteration of *in vivo* bone collagen isotope ratios in relation to palaeodietary reconstruction, *Nature*, **317**(**6040**), 806-809.

Guiry, E. J., P. Szpak, and M. P. Richards, 2016, Effects of lipid extraction and ultrafiltration on stable carbon and nitrogen isotopic compositions of fish bone collagen, *Rapid Communications in Mass Spectrometry*, **30**(**13**), 1591-1600.

Magnusson, B., T. Näykki, H. v. Hovind, and M. Krysell, 2012, Handbook for Calculation of Measurement Uncertainty in Environmental Laboratories, Nordtest Technical Report 537 ed. 3.1.

Post, D., C. Layman, D. Arrington, G. Takimoto, J. Quattrochi, and C. Montaña, 2007, Getting to the fat of the matter: models, methods and assumptions for dealing with lipids in stable isotope analyses, *Oecologia*, **152**(**1**), 179-189.

Qi, H., T. B. Coplen, H. Geilmann, W. A. Brand, and J. K. Böhlke, 2003, Two new organic reference materials for δ^13^C and δ^15^N measurements and a new value for the δ^13^C of NBS 22 oil, *Rapid Communications in Mass Spectrometry*, **17**(**22**), 2483-2487.

Qi, H., T. B. Coplen, S. J. Mroczkowski, W. A. Brand, L. Brandes, H. Geilmann, and A. Schimmelmann, 2016, A new organic reference material, l-glutamic acid, USGS41a, for *δ*^13^C and *δ*^15^N measurements − a replacement for USGS41, *Rapid Communications in Mass Spectrometry*, **30**(**7**), 859-866.

Szpak, P., 2011, Fish bone chemistry and ultrastructure: implications for taphonomy and stable isotope analysis, *Journal of Archaeological Science*, **38**(**12**), 3358-3372.

Szpak, P., K. Krippner, and M. P. Richards, 2017a, Effects of Sodium Hydroxide Treatment and Ultrafiltration on the Removal of Humic Contaminants from Archaeological Bone, *International Journal of Osteoarchaeology*, **27**(**6**), 1070-1077.

Szpak, P., J. Z. Metcalfe, and R. A. Macdonald, 2017b, Best Practices for Calibrating and Reporting Stable Isotope Measurements in Archaeology, *Journal of Archaeological Science: Reports*, **13**, 609-616.

van Klinken, G. J., and R. E. M. Hedges, 1995, Experiments on Collagen-Humic Interactions: Speed of Humic Uptake, and Effects of Diverse Chemical Treatments, *Journal of Archaeological Science*, **22**(**2**), 263-270.

Supplementary Table S5

Mapping coverage information. The genomic data were mapped to the mitochondrial reference genomes of beluga (KY444734) and narwhal (NC_005279), and the nuclear genome of killer whale (GCA_000331955.2).

Supplementary Figure S6

Median-spanning haplotype network of complete mitochondrial genomes of eight belugas (blue), eight narwhals (green) and MCE1356 mapped to the beluga reference (red). Black dots indicate intermediate haplotypes not found in the data. The size of the circles indicate the relative number of specimens sharing a haplotype. Numbers indicate number of variable sites between haplotypes.


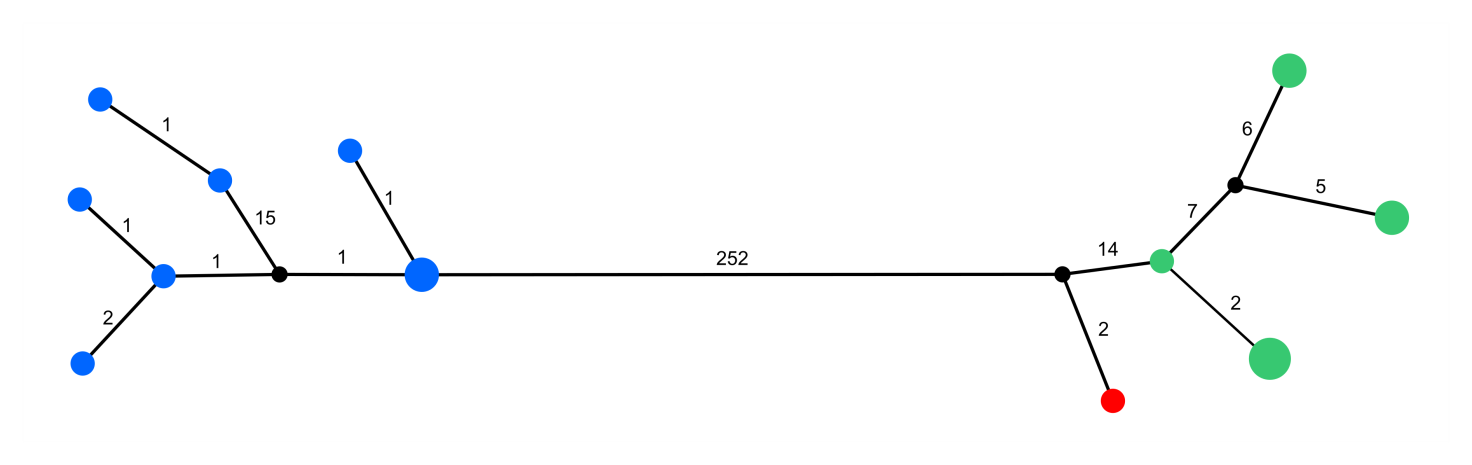


Supplementary Figure S7

Combined read depth distribution of MCE1356, eight belugas and eight narwhals. Mean value is 4.14.


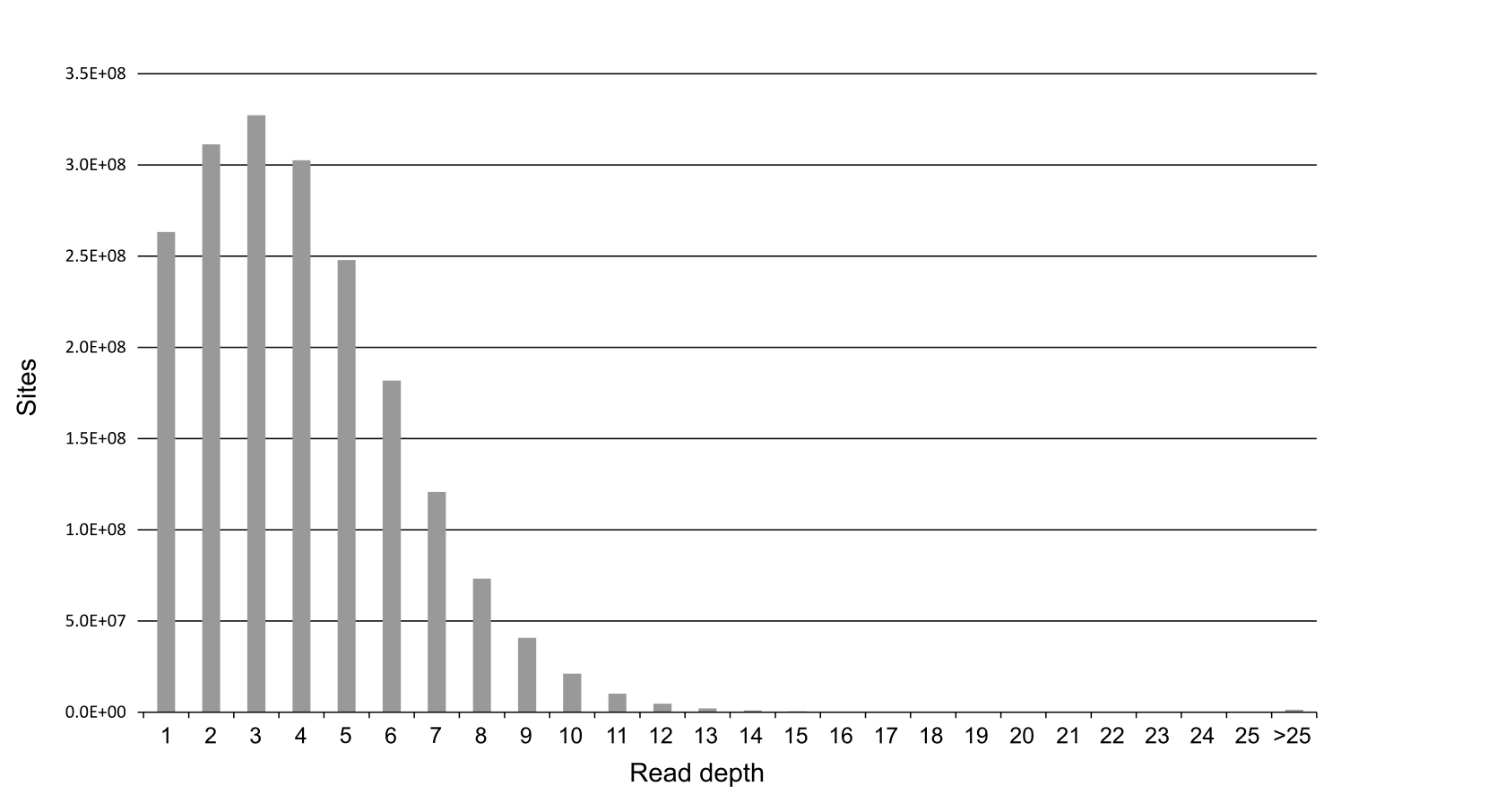

Supplement: Supplementary file 1 — Hybridization between Arctic cetaceans Supplementary [file 41598_2019_44038_MOESM1_ESM.docx]
